# YOLO-OD: Obstacle Detection for Visually Impaired Navigation Assistance

**DOI:** 10.3390/s24237621

**Published:** 2024-11-28

**Authors:** Wei Wang, Bin Jing, Xiaoru Yu, Yan Sun, Liping Yang, Chunliang Wang

**Affiliations:** College of Computer Science and Technology, Changchun University, No. 6543, Satellite Road, Changchun 130022, China; 231502527@mails.ccu.edu.cn (B.J.); 231501508@mails.ccu.edu.cn (X.Y.); 221501464@mails.ccu.edu.cn (Y.S.); yangliping@ccu.edu.cn (L.Y.); 221501491@mails.ccu.edu.cn (C.W.)

**Keywords:** obstacle detection, YOLO-OD, feature weighting block, adaptive bottleneck block, enhanced feature attention head

## Abstract

Visually impaired individuals frequently encounter difficulties in detecting and avoiding obstacles in the wild. To address this issue, we propose an obstacle detection method for visual navigation assistance, named YOLO-OD. To improve the ability to detect and differentiate between different sized obstacles in outdoor environments, we introduce the Feature Weighting Block (FWB), which improves feature importance discrimination. To address the challenges of detecting cluttered outdoor environments and handling occlusions, we introduce the Adaptive Bottleneck Block (ABB), which captures varying features across different scenes. To solve the problem of detecting relatively small obstacles in outdoor environments, we propose the Enhanced Feature Attention Head (EFAH). The proposed YOLO-OD achieves an average precision of 30.02% on a public dataset, making it a worth studying approach for blind and visually impaired navigation aids.Our study effectively addresses the navigation challenges faced by visually impaired individuals by improving model performance, thereby enhancing its practical values. The code for YOLO-OD has been made publicly available to ensure reproducibility and facilitate further research.

## 1. Introduction

According to the World Health Organization, the global population is approximately 7.7 billion, with around 246 million individuals experiencing visual impairments, including 39 million who are blind. A primary challenge faced by visually impaired individuals is mobility, as they struggle with recognizing and navigating obstacles, particularly in urban environments. These obstacles significantly impact their safety and independence. Advances in computer technology have led to a proliferation of computer-assisted devices, particularly in image processing and medical image analysis. However, visual detection tasks for visually impaired individuals are particularly hindered by challenges such as occlusion [1], dense crowds [2], and small object detection [3], especially in real-world settings with complex environments. These challenges limit the effectiveness of existing assistive technologies, making it difficult for visually impaired individuals to navigate safely. Occlusion complicates object localization, while small objects are challenging to detect due to their diminutive size and inconspicuous features. In densely populated scenes, overlapping objects blur boundaries, further complicating segmentation and recognition. Accurate and efficient object detection technology is crucial for visually impaired individuals. However, existing algorithms often struggle with precision and reliability in practical applications, particularly when faced with overlapping objects, varying lighting conditions, and small obstacle detection. These limitations make current solutions insufficient for ensuring the safety and autonomy of visually impaired individuals. Therefore, the research presented in this paper is essential for addressing these challenges.

Visually impaired individuals need to locate and avoid obstacles in strange environments [4]. Traditional techniques, such as guide dogs [5] and white canes [6], are limited in terms of speed, precision, and coverage. Guide dogs are effective navigation means, but they cannot detect every small obstacle and are also costly and require professional training. White canes, on the other hand, can only detect obstacles within a very limited range, making them insufficient for safe navigation in complex urban settings. Consequently, there is an urgent need for computer-aid approaches [7]. Numerous studies utilizing object detection aim to assist visually impaired individuals. For example, Said et al. [8] proposed a real-time intelligent navigation assistance system. Compared to traditional methods, data-driven object detection technologies offer higher precision, greater generalizability, and sustainable development potential. These advances have the potential to be integrated into assistive devices, providing visually impaired individuals with a greater sense of safety and independence in navigating urban and unfamiliar environments.

In the field of computer vision, object detection is a fundamental task that has been extensively researched. Object detectors can be roughly defined as one-stage and two-stage methods. One-stage object detectors [9,10,11] perform both object localization and classification by directly predict bounding boxes and class scores for each object from extracted features. These methods are generally faster as they perform detection and classification in a single network pass, which makes them well-suited for real-time applications. In contrast, two-stage object detectors [12,13,14] employ a two-step strategy. These detectors first generate region proposals in the first stage and then refine these proposals by classifying them and adjusting their bounding boxes in the second stage. These methods normally offer higher detection precision and generalizability, which makes them ideal for scenarios that demand high precision. The advent of universal object detectors has facilitated the deployment of object detection technology in assistive devices designed for individuals with visual impairments. By enabling real-time recognition of pedestrians, vehicles, and other obstacles, object detection helps visually impaired individuals navigate more effectively and improves their quality of life.

**Figure 1:**
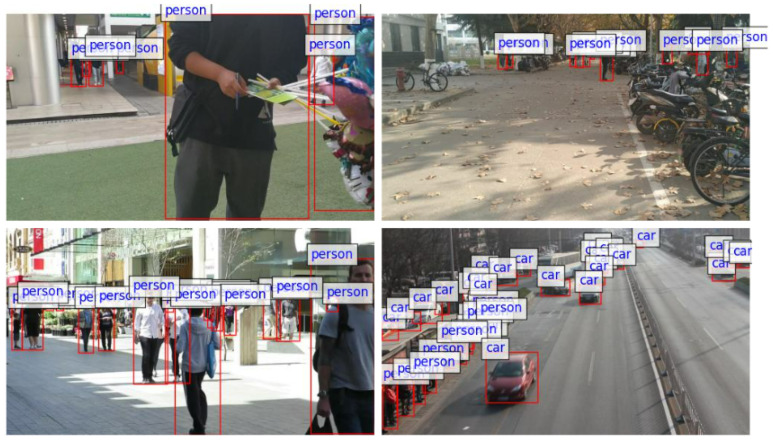
Visualization of picture samples from the real world.

As illustrated in Figure 1, for visually impaired individuals, object detection in the real world often suffers from challenges such as occlusion, small object detection, and complex backgrounds, making safe navigation extremely challenging in dynamic environments. Specifically, occlusion results in an incomplete presentation of object features, which can impair semantic information and increase the difficulty of subsequent regression and classification, directly impacting the ability of visually impaired individuals to accurately detect obstacles in their surroundings, especially in busy environments. The high complexity of densely crowded scenes, such as crowded pedestrian crossings, poses a significant challenge to feature extractors because distinguishing between objects and backgrounds becomes difficult, leading to redundant information that interferes with object features. This significantly affects the ability of assistive technologies to provide timely and accurate information for visually impaired individuals. Overlapping object regions also present a challenge to detectors trying to distinguish between different object boundaries. In the detection of smaller obstacles, such as potholes or traffic cones, due to the lack of effective pixels representing these smaller obstacles, less useful information is extracted, which ultimately hampers the effectiveness of traditional detection methods. This issue is especially critical for visually impaired individuals who rely on precise detection of small obstacles to ensure their safety. This hampers the ability of feature extractors to accurately adapt to the features of objects across different scales, ultimately limiting the discriminative power and precision of the features. Our proposed methods aim to mitigate these challenges by enhancing feature extraction and improving robustness to occlusion and background complexity, ultimately ensuring that visually impaired individuals can receive reliable and accurate assistance in various real-world environments.

To address the aforementioned challenges, we propose YOLO-OD. Building on YOLOv8, we integrated an Adaptive Bottleneck Block (ABB) within the detection neck to enhance object detection in complex scenes, added a Feature Weighting Block (FWB) to process features initially extracted by the backbone, Enhanced Feature Attention Head (EFAH) is proposed, which incorporates Convolutional Block Attention Module (CBAM) [15] into the classification header to improve the classification precision, and introduces a transposed convolution layer and a convolutional layer in the regression header to improve the localization precision. These modifications collectively aim to enhance the robustness and precision of the model, thereby providing effective navigational assistance to visually impaired individuals.

To provide a concise overview of our contributions, we present them in five key aspects:

(1) We propose a new obstacle detection method that incorporates a feature-weighted block (FWB), an adaptive bottleneck block (ABB), and an enhanced feature attention head (EFAH) to improve usability in visual impairment detection.

(2) To enhance the detection and distinction of obstacles of varying sizes in outdoor environments, we introduce the Feature Weighting Block (FWB), designed to improve the discrimination of feature importance.

(3) To tackle the challenges of cluttered outdoor environments and occlusion, we present the Adaptive Bottleneck Block (ABB), which captures diverse features across different scenes.

(4) For the detection of relatively small obstacles, We propose the Enhanced Feature Attention Head (EFAH), which can better capture the detailed information of features through the transposed convolution layer.

(5) Our proposed method was validated on a publicly available dataset and achieved an average accuracy of 30.02%, making it a worthwhile research method for navigational aids for the blind and visually impaired.

## 2. Related Work

### 2.1. One-Stage Detectors

One-stage object detectors, exemplified by the YOLO [16] and SSD series, are optimized for real-time applications due to their high detection speeds. YOLOv3 [17] introduced a more complex network structure and multi-scale predictions, thereby improving precision while maintaining speed. YOLOv4 [18] further optimized the architecture with CSPNet and various training techniques, enhancing both speed and precision. YOLOv5 [19] was designed to enhance ease of use, with a focus on ensuring efficient implementation and deployment. One of the state of the art YOLO, YOLOv8 [20], introduces architectural refinements, enhanced anchor-free detection, dynamic task prioritization, and superior real-time performance.The Single Shot MultiBox Detector SSD [21] utilize multi-scale feature maps for efficient object detection, offering a balance between speed and accuracy without region proposal steps, ideal for real-time applications.

### 2.2. Two-Stage Detectors

Two-stage object detectors, particularly the R-CNN series [22], are distinguished by their precision in object detection. R-CNN (Region-CNN) [23] introduces the two-stage approach by generating region proposals using selective search and applying CNNs to classify each proposal. While effective, it was slow due to the need for individual CNN processing per region. Fast R-CNN [24] improves the efficiency of R-CNN by using shared convolutional feature maps and introducing RoI pooling. This allowed all region proposals to share the same feature map, greatly reducing computation time and maintaining high accuracy. Faster R-CNN [25] introduces the Region Proposal Network (RPN), integrating proposal generation directly into the network, which significantly increased speed and efficiency. By jointly predicting object bounds and scores, it streamlined the two-stage detection process.Cascade R-CNN [26] extendes the Faster R-CNN architecture by progressively refining region proposals across multiple stages, with each stage using increasingly higher IoU thresholds. This made the model more accurate for difficult detection cases, especially in handling high-quality proposals.

### 2.3. Similar Works

Many researchers have already done similar work on solving the problem of obstacle avoidance for the visually impaired [27,28]. Atitallah et al. [29] propose developing an obstacle detection system based on a modified YOLOv5 neural network architecture. The proposed system is capable of recognizing and locating a set of indoor and outdoor objects that are extremely useful for Blind and Visually Impaired (BVI) navigation aids. Woo et al. [30] propose a smart data driven mobility aid for VIP (SOMAVIP), enabling real-time scene perception. The ODOMOVIP comprises of responsive devices and IoT embedded smart city environments, deep machine learning, computer vision, and data processing methods. Although much research has been conducted, many challenges remain, such as the significant shortcomings in detecting small objects and complex backgrounds. Our proposed YOLO-OD addresses these issues by utilizing FWB for preliminary weighting of the extracted features, ABB to enhance the ability of model to detect objects in complex scenes, by adding multi-scale convolution, learnable normalization layer and learnable activation function. Finally, EFAH to improve the classification and regression of model by capturing more detailed information.

## 3. Methodology

### 3.1. Architecture of the Proposed Method

**Figure 2:**
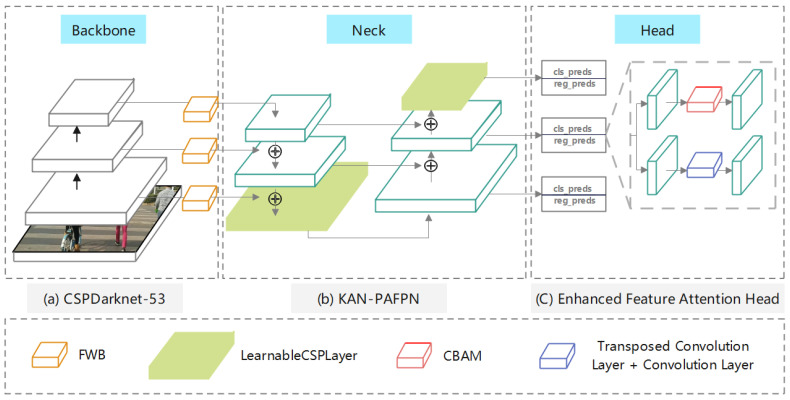
Architecture of the proposed method.

To address the challenges faced by visually impaired individuals in complex environments, such as small obstacles, occlusion, and background noise, we propose YOLO-OD, inspired by the architecture of YOLOv8. As illustrated in Figure 2, the proposed YOLO-OD leverages its advanced structure to improve detection performance, particularly in real-world tests involving urban obstacles such as potholes, traffic cones, and pedestrian congestion. These enhancements are crucial for improving the safety and independence of visually impaired individuals. The YOLO-OD consists of three main components: the backbone network (CSPDarknet-53), the detection neck (KAN Path Aggregation Feature Pyramid Network, KANPAFPN) [31], and the detection head (Enhanced Feature Attention Head).

CSPDarknet-53 is chosen as the backbone due to its effective utilization of cross-stage partial connections, which scientifically optimize feature extraction by reducing redundancy in the network. This structure allows for efficient feature reuse and enhances the ability of model to process high-dimensional data with minimal computational overhead. The multiscale feature extraction capabilities of backbone make it particularly well-suited for identifying both subtle and prominent obstacles, essential for assisting visually impaired individuals in dynamic environments.

The KAN-PAFPN is employed for feature fusion, where integration is executed through top-down and bottom-up pathways to capture detailed and abstract features. However, the indiscriminate merging of features may cause significant information to be diluted, especially in urban environments where visually impaired individuals need precise obstacle detection. Subsequently, the extracted features from the obstacles are indiscriminately merged within the PAFPN, resulting in equal treatment of both significant and insignificant features, the model may struggle to prioritize key information, potentially leading to missed detections or inaccurate classifications. To address this challenge, the incorporation of the Feature Weighting Block (FWB) before the KAN-PAFPN allows for differential weighting of features, which significantly improved the ability of model to detect small and critical obstacles in real-world tests, such as potholes and narrow pathways, ensuring more reliable navigation for visually impaired individuals [32]. Furthermore, to address the complex environments faced by visually impaired individuals, we introduces the Adaptive Bottleneck. The module enhances object detection by capturing diverse features and adapting to various scenarios, thereby improving the overall precision and robustness of the system.

The EFAH consists of two parts: the classification head and the regression head. The classification head incorporates the CBAM, which selectively focuses on spatial and channel-wise information, helping to differentiate obstacles more effectively from complex backgrounds. To enhance performance in scenarios involving visually impaired individuals, several modifications have been implemented. The classification head incorporates the CBAM to enhance its ability to distinguish between obstacles. The regression head has been enhanced with a transposed convolution followed by a convolution layer, adds more detailed information, improves precision in predicting obstacle locations for more reliable navigation assistance [33].

### 3.2. Feature Weighting Block

**Figure 3:**
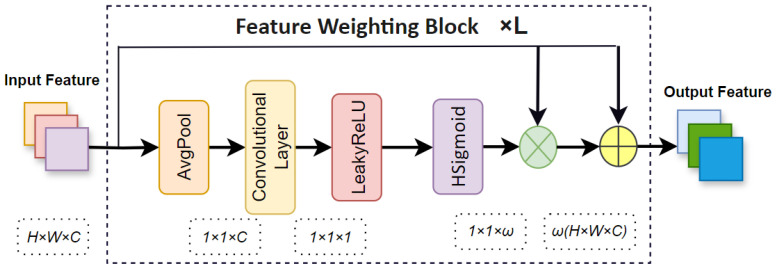
Feature Weighting Block.

As illustrated in Figure 3, to help visually impaired individuals navigate safely, particularly in urban environments with varying obstacles, we propose the FWB, which is a feature-weighted processing module. The specific computational process can be described as follows:

(1) $$\begin{array}{cc} F & =FWB(f)\\ FWB(f) & =HSigmoid(LeakyReLU(Conv2d\_1\times 1\\ \; & \;(AvgPool(f)))) \end{array}$$

where *f* represents the original features extracted by CSPDarknet-53, with dimensions H×W×C, where *H*, *W*, and *C* represent the height, width, and number of channels, respectively. Conv2d_1×1 represents a convolution layer with a feature kernel size of 1 × 1, mainly used to adjust the number of channels. Averagepooling (Avgpool) is a downsampling technique in convolutional neural networks that reduces the spatial dimensions of feature maps by taking the average of values within a sliding window, after which the features become 1×1×C, Following this, a convolutional layer adjusts the number of channels to 1. The LeakyReLU activation function is mainly used to apply a small non-zero slope to negative input values to ensure that all inputs contribute to the output, thus preventing neurons from becoming inactive during training [34]. The Hard Sigmoid activation function is a computationally efficient piecewise linear approximation of the standard sigmoid, designed to reduce complexity by capping and scaling inputs without the use of exponential computation, after which values between 0 and 1 are compressed, producing an output dimension of 1×1×w, where *w* is a specific value. *F* is the final feature representation. FWB assigns weights to said features based on their semantic importance, ensuring that more critical features related to obstacles are highlighted. This is crucial for improving detection accuracy in scenarios where small or partially occluded obstacles might be overlooked by conventional methods. This block allows the model to better differentiate between obstacles of different sizes, as demonstrated in our experiments with datasets containing varying-sized obstacles. In particular, the FWB showed a significant improvement in detecting smaller obstacles, which are often missed by conventional models. This enhancement is crucial in providing reliable navigational assistance to visually impaired individuals in complex and dynamic environments.

### 3.3. Adaptivebottleneck Block

**Figure 4:**
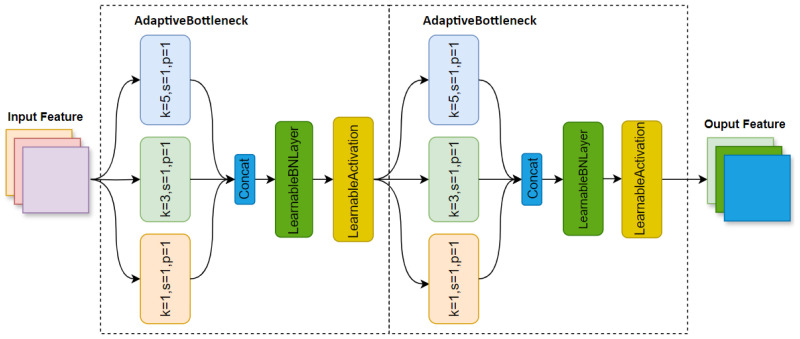
AdaptiveBottleneck Block.

As illustrated in Figure 4, we propose the Adaptive Bottleneck Block in KAN-PAFPN, which aims to address the lack of flexibility when dealing with various scenarios and features. The Adaptive Bottleneck Block enhances the ability of model to detect high-contrast edges and fine textures in complex environments. This capability is crucial for providing safer and more reliable obstacle avoidance assistance for visually impaired individuals navigating urban environments. To achieve this, the module employs multi-scale convolutional kernels (1 × 1, 3 × 3, 5 × 5) [35] to capture multi-scale features. The use of multi-scale convolutional kernels allows the model to capture diverse features across different receptive fields, which is particularly important for detecting a wide range of obstacles, from large, easily visible barriers to small, less noticeable hazards that visually impaired individuals might encounter on urban streets. The multi-scale convolutional kernel is calculated as follows:

(2) $$\begin{array}{cc} f_{1\times 1} & =Conv2d_{1\times 1}\left(f\right)\\ f_{3\times 3} & =Conv2d_{3\times 3}\left(f\right)\\ f_{5\times 5} & =Conv2d_{5\times 5}\left(f\right) \end{array}$$

(3) $$f_{concat}=Concat\left(f_{1\times 1},f_{3\times 3},f_{5\times 5}\right)$$

where $ConvLayer_{n\times n}$ refers to a convolution layer with a kernel size of n×n. Concat means the features are concatenated along the channel dimension. After the convolutionallayers, the fixed BatchNormalizationlayer is replaced by the LearnableBatchNormalizationlayer, which includes additional trainable parameters that adaptively adjust the normalization process based on the input data distribution.

In the standard Batch Normalization (BN) layer, the normalized output is computed as:

$$\hat{x}=\frac{x-\mu_{\mathrm{batch}}}{\sqrt{\sigma_{\mathrm{batch}}^{2}+\epsilon }}$$

where $\mu_{\mathrm{batch}}$ and $\sigma_{\mathrm{batch}}^{2}$ are the mean and variance calculated across the batch, and ϵ is a small constant for numerical stability, $\hat{x}$ indicates the output eigenvalues after normalization. Here, the mean and variance are calculated statically from the current batch of data, limiting the model’s ability to adapt to more complex data distributions over time.

Next, a linear transformation is applied:

$$y=\gamma \hat{x}+\beta$$

where *y* represents the output eigenvalue after normalization and linear transformation. γ and β are trainable parameters that scale and shift the normalized features. This allows the model to adjust the normalized features slightly, but the reliance on batch statistics for $\mu_{\mathrm{batch}}$ and $\sigma_{\mathrm{batch}}^{2}$ makes it less flexible in handling diverse or complex input distributions.

However, in our proposed LearnableBatchNormlayer, we introduce additional flexibility by making the mean and variance learnable. This is achieved by introducing trainable parameters $\Delta_{\mu }$ and $\Delta_{\sigma }$, which allow the model to adjust the mean and variance during the training process, rather than relying solely on batch statistics. The normalization process is calculated as:

$$f_{norm}=LearnableBatchNormLayer\left(\gamma \frac{x-(\mu_{\mathrm{batch}}+\Delta_{\mu })}{\sqrt{\sigma_{\mathrm{batch}}^{2}+\Delta_{\sigma }+\epsilon }}+\beta \right)$$

where $\Delta_{\mu }$ and $\Delta_{\sigma }$ are trainable parameters that adjust the batch mean and variance, respectively. This modification allows the normalization process to dynamically adjust based on the varying input data distribution, such as different lighting conditions in urban areas or varying obstacle textures in crowded environments. Experiments show that this approach enhances the detection stability in these challenging scenarios, thereby improving the safety of visually impaired individuals.

By introducing these learnable adjustments, $\Delta_{\mu }$ and $\Delta_{\sigma }$, the Adaptive Batch Normalization module provides the flexibility to handle diverse input conditions more effectively than standard Batch Normalization. This capability is especially useful in visually impaired navigation scenarios where environmental conditions, such as lighting and obstacle types, can vary significantly. Specifically:

- $\Delta_{\mu }$ acts as a learnable shift to the batch mean $\mu_{\mathrm{batch}}$, allowing the model to modify the mean in response to changes in data distribution.
- $\Delta_{\sigma }$ adjusts the batch variance $\sigma_{\mathrm{batch}}^{2}$, allowing the model to fine-tune the spread of the data distribution.

Thus, the learnable parameters γ, β, $\Delta_{\mu }$, and $\Delta_{\sigma }$ enable the LearnableBatchNormlayer to adapt more effectively to diverse input conditions, making it particularly useful in complex and dynamic environments, such as obstacle detection for visually impaired individuals, where robustness is critical for system performance.

The learnable adjustments to mean and variance provide greater stability and adaptability, especially when working with small batch sizes or data with high variability, resulting in improved model robustness.

This adaptive normalization technique enhances robustness in a variety of situations, including different lighting conditions and complex backgrounds, and thus effectively helps visually impaired people with obstacle avoidance. In addition, the AdaptiveBottleneck module incorporates the LearnableActivation function. Unlike fixed activation functions such as SiLU in the original pafpn, the LearnableActivation function is calculated as:

(4) $$F=LearnableActivation(\omega \left(\frac{x}{1+e^{-x}}+\sum_{i}c_{i}B_{i}\left(x\right)\right))$$

where *F* represents the output feature map after processing through the Adaptive Bottleneck module, ω and $c_{i}$ being trainable parameters, *x* represents the feature, and $B_{i}\left(x\right)$ representing basis functions such as B-splines.

This dynamic adjustment ensures optimal non-linear transformation for varying input features, enhancing the detection of high-contrast edge features and fine textures, which are critical for accurate obstacle detection.

### 3.4. Enhanced Feature Attention Head

**Figure 5:**
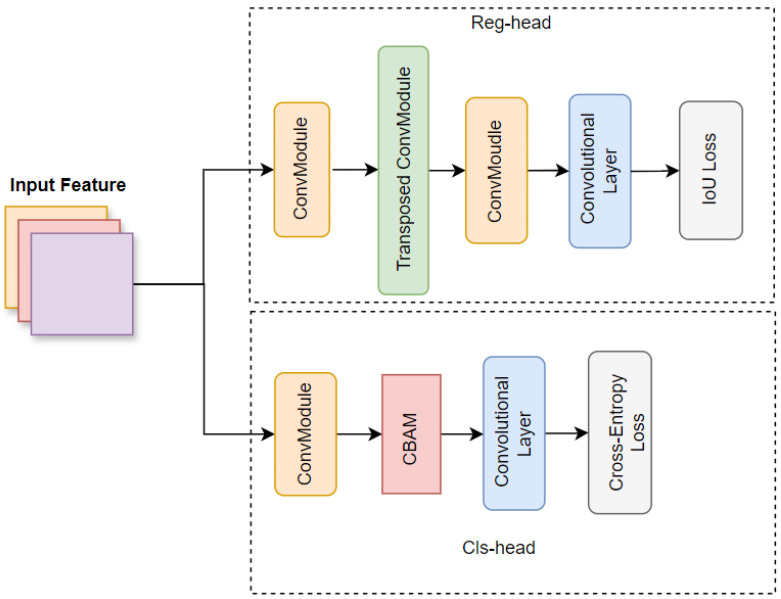
Enhanced Feature Attention Head.

As illustrated in Figure 5, in order to improve the detection accuracy of smaller obstacles in scenarios where visually impaired people are traveling, we propose the Enhanced Feature Attention Head, which is divided into a classification head and a regression head. In the classification stage, the input feature is first processed through a convolutional layer, which extracts preliminary features. Subsequently, the Convolutional Block Attention Module (CBAM) is integrated to refine the feature by focusing on crucial regions and suppressing irrelevant information. This attention mechanism enhances the information on space and channel, thereby improving classification accuracy. A subsequent convolutional layer operation processes these attended features, which are then utilized to calculate the classification loss. In the regression head, the input feature is firstly processed by a convolutional layer to resize and channel dimension. For precise localization, a transposed convolution module is introduced to expand the size of the features so that more detailed information about the features can be captured, thus helping to achieve finer object localization. This is followed by another convolutional layer that refines the upsampled features, resize features back to original size. A final convolutional layer processes these refined features, which are then used to output prediction results.

## 4. Experiment

### 4.1. Datasets and Evaluation Metrics

The dataset used in this article is a road obstacle dataset in COCO format, available at the link [36]. This dataset contains a total of 6276 images, which has four categories, with the number of samples for each category as follows: car: 22,318, person: 53,756, traffic cone: 1176, and pothole: 1719.

We employ a set of metrics to evaluate the effectiveness of our model, including mAP (50, s, m, l), GFLOPS, and Params. mAP is the Average Precision (AP) across all classes. mAP50 represents the mean average precision at an IoU (Intersection over Union) threshold of 0.5. mAP_*s*_ quantifies the mean detection precision for objects with an area smaller than 32 × 32 pixels, mAP_*m*_ assesses the mean detection precision for objects with an area between 32 × 32 and 96 × 96 pixels, and mAP_*l*_ evaluates the mean detection precision for objects with an area larger than 96 × 96 pixels. GFLOPS is employed to evaluate the computational speed of a method, whereas Params is utilized to quantify the number of parameters within the method.

### 4.2. Implementation Details

The code for the proposed model and the comparison models was implemented using the MMYOLO and MMDetection frameworks. YOLO-OD was trained on two RTX 3090 GPUs for 150 epochs, while the other models used their default settings. MMDetection used the SGD optimizer with a learning rate of 0.02, while MMYOLO used the SGD optimizer with a learning rate of 0.01. For MMDetection, the input image size was 1333 × 800 pixels, and the test image size was also 1333 × 800 pixels. In contrast, MMYOLO used an input image size of 640 × 640 pixels. The batch size was set to 16 by default, and the confidence threshold for visualization was set to 0.5 by default.

### 4.3. Ablation Studies

Effect of the Feature Weighting Block (FWB). In Table 1, the addition of FWB to YOLOv8-s leads to a modest increase in computational cost, with the parameters rising from 11.13 to 11.14 and GFLOPs from 14.27 to 14.28. However, the benefits are evident through the improvement in mAP_*s*_, which increases from 18.93 to 20.02. This gain in performance demonstrates the effectiveness of FWB in enhancing small obstacle detection capabilities, which is particularly crucial for visually impaired individuals navigating complex environments. Furthermore, there is also a significant improvement in overall mAP, increasing from 28.51 to 29.33, mAP_*m*_ from 28.93 to 30.42, and mAP_*l*_ from 34.01 to 34.72. These improvements indicate that FWB not only provides considerable enhancements in small object detection but also yields notable gains in the detection of medium and large objects. This comprehensive enhancement across different object sizes underscores the versatility of FWB in improving the overall detection performance of the model. Similarly, Table 2 demonstrates the impact of incorporating FWB into YOLOv5-s. The parameters increase slightly from 8.12 to 8.13, and GFLOPs rise from 12.33 to 12.34. Despite this minimal increase, there is a significant improvement in the mAP_*s*_ metric, which rises from 11.24 to 12.17. Additionally, the overall mAP improves from 14.23 to 15.22, mAP_*m*_ from 17.71 to 19.14, and mAP_*l*_ from 22.88 to 24.55. These improvements clearly demonstrate that the inclusion of FWB not only enhances the ability of model to detect small obstacles but also significantly improves its performance for medium and large objects. This further validates the effectiveness of FWB across different scenarios and object scales. The inclusion of FWB significantly enhances the feature representation capability of the model, thereby achieving substantial improvements in detection performance with a negligible impact on model complexity.

Effect of the Adaptive Bottleneck Block (ABB). As shown in Table 1, adding the Adaptive Bottleneck Block (ABB) significantly improves YOLOv8-s in terms of both small and medium obstacle detection. Specifically, mAP_*s*_ increased from 18.93 to 20.23, and mAP_*m*_ rose from 28.93 to 29.94. Although the computational cost increased—with GFLOPs rising from 14.27 to 58.52 and parameters growing from 11.14 to 40.09—this trade-off is justified by the notable accuracy gains. Such improvements are crucial for accurately detecting overlapping and small obstacles in complex urban environments, thereby enhancing the safety of navigation systems for visually impaired individuals. Similarly, Table 2 shows that incorporating ABB into YOLO X-s increased the mAP from 23.22 to 24.76 and mAP_50_ from 40.31 to 41.76. While the GFLOPs increased from 13.32 to 47.92 and the parameters grew from 8.94 to 38.56, these performance improvements highlight effectiveness of ABB in capturing complex features. This enhanced capability is particularly beneficial in navigating intricate and cluttered environments, offering more reliable detection of potential obstacles, which is key to supporting visually impaired individuals.

Effect of the Enhanced Feature Attention Head (EFAH). As shown in Table 1, integrating the Enhanced Feature Attention Head (EFAH) into YOLOv8-s results in improved performance across various metrics, with mAP increasing from 28.51 to 29.44, mAP_*s*_ rising from 18.93 to 20.23, and only a slight increase in model complexity, as GFLOPs grow from 14.27 to 14.90 and parameters from 11.14 to 11.37. This indicates that EFAH effectively enhances the ability of model to focus on crucial features, significantly benefiting the detection of small obstacles, which is critical for navigation assistance for visually impaired individuals in crowded environments. In Table 3, applying EFAH to Deformable DETR yields similar advantages, with mAP_*s*_ improving from 15.92 to 16.73 and mAP_*l*_ from 30.36 to 31.65. Although GFLOPs and parameters increase from 208.00 to 231.00 and 41.36 to 41.63, respectively, this slight complexity boost is offset by the enhanced detection accuracy. Such improvements are essential in complex scenarios involving obstacles of various sizes, ensuring the model can capture fine details necessary for the safe and efficient navigation of visually impaired individuals. The consistent gains from EFAH across different models underscore its effectiveness in addressing the challenge of small-object detection, providing an essential enhancement for applications requiring precise obstacle recognition in dynamic and intricate environments.

### 4.4. Comparisons

As illustrated in Table 4, the proposed method demonstrates a significant advantage over both the YOLO series models [20] and other mainstream object detectors, including the one-stage SSD [21] model and two-stage detectors like Faster R-CNN [25], Cascade R-CNN [26], and Deformable DETR [37]. This superiority is evident in both accuracy and efficiency, especially in detecting small objects—a critical feature for ensuring the safe navigation of visually impaired individuals in complex environments. Compared to the baseline YOLOv8-s model, our method achieves an increase in mAP from 28.51 to 30.01 and in mAP_*s*_ from 18.93 to 20.96, underscoring the enhanced ability of model to detect small, less conspicuous obstacles. Such improvements are crucial for applications requiring precise small-object detection, as they provide better obstacle awareness in environments where visual cues are limited or subtle. Additionally, while the two-stage Cascade R-CNN achieves a respectable mAP of 27.23 and mAP_*s*_ of 17.12, our model surpasses it with 30.01 and 20.96, respectively, while also being more computationally efficient. Specifically, our model has fewer parameters (48.32 vs. 69.16) and lower GFLOPs (59.15 vs. 236.00), making it more suitable for deployment on resource-limited assistive devices. The proposed method stands out as the most precise among both the two-stage detectors (Faster R-CNN, Cascade R-CNN, Deformable DETR) and the one-stage detectors (SSD, YOLOv5-s, YOLOv7-tiny, YOLOX-s, YOLOv8-s, YOLOv10-n). Its ability to excel in detecting small obstacles further substantiates its potential as an effective tool for assistive technology aimed at visually impaired individuals. Overall, the proposed model’s high precision, combined with its efficient computation, positions it as a robust solution for developing assistive technology dedicated to enhancing the independence and safety of visually impaired users. The performance gains in small-object detection, in particular, highlight the capability of model to navigate complex urban environments, making it an invaluable aid in supporting visually impaired individuals in real-world scenarios.

### 4.5. Visualization

**Figure 6:**
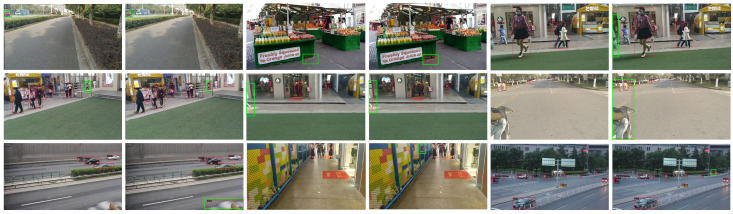
Visualization of YOLOv8 (**left**) and YOLO-OD (**right**) detection results.

As illustrated in Figure 6, the detection results of YOLOv8 (left) and our proposed YOLO-OD (right) are visualized. The red box represents the actual detected box, and the green box highlights the difference between the detection results of the two models. We can see that the red car in the first row on the left is not detected by YOLOv8, whereas it is accurately detected by YOLO-OD, reflecting the superiority of YOLO-OD in detecting small obstacles that are crucial for visually impaired individuals. Similarly, the pedestrians in the first set of images in the second row are not detected by YOLOv8 due to the occlusion of the railings and the complex background. However, they are accurately detected by YOLO-OD, demonstrating the ability of YOLO-OD to handle challenging conditions such as occlusion and complex environments. This ability is particularly critical for visually impaired individuals who need reliable detection to navigate safely through urban areas with many obstacles. These visualization results clearly illustrate the practical benefits of YOLO-OD in real-world scenarios, particularly for visually impaired individuals who face significant challenges when navigating through environments with multiple overlapping objects and varying background complexities.

## 5. Discussion

Our work proposes an object detection method named YOLO-OD, specifically designed to help visually impaired individuals navigate safely through challenging environments, such as low-light or cluttered urban areas, by effectively detecting obstacles that may compromise their mobility and safety. YOLO-OD was developed to address the shortcomings of existing object detection methods, which often struggle with small or occluded obstacles in dynamic environments that visually impaired individuals face. We have demonstrated the superior detection performance of YOLO-OD through experiments on public obstacle detection datasets, emphasizing its capability to overcome these challenges. The experimental results show that YOLO-OD significantly outperforms other comparison models, particularly in detecting small and partially occluded obstacles which are critical for the safety of visually impaired individuals. We conducted ablation experiments on different models, including both one-stage and two-stage detectors, to understand the contribution of individual components, such as the Adaptive Bottleneck Block and Feature Weighting Block, to the improved performance of YOLO-OD in challenging environments. Our goal is to further improve the detection accuracy of our method while optimizing inference speed. Below are the directions for improvement that we plan to explore:

1. Addressing the specific challenges of obstacle detection for visually impaired individuals in low-light conditions to further refine the model.

2. Upgrading the model using techniques such as wavelet convolution and StarNet to enhance both detection accuracy and inference speed.

Through these improvements, we expect that YOLO-OD will achieve even more outstanding performance in obstacle detection tasks for visually impaired individuals, providing stronger support for those with visual impairments.

## Figures and Tables

**Table 1:** Ablation experiments.

Models	mAP	mAP_50_	mAP_*s*_	mAP_*m*_	mAP_*l*_	Flops/G	Parameters/M
YOLOv8-s (baseline)	28.51	42.02	18.93	28.93	34.01	14.27	11.14
YOLOv8-s + FWB	29.33	42.54	20.02	30.42	34.72	14.28	11.14
YOLOv8-s + ABB	29.55	42.85	20.23	29.94	35.14	58.52	40.09
YOLOv8-s + EFAH	29.44	42.46	19.25	29.86	35.06	14.90	11.37
YOLOv8-s + FWB + ABB	30.00	42.33	20.87	30.41	35.74	58.53	48.09
YOLOv8-s + ABB + EFAH	29.78	42.86	19.98	30.05	35.37	59.14	48.32
YOLOv8-s + FWB + EFAH	29.72	42.72	19.92	30.35	35.08	14.90	11.37
YOLOv8-s + FWB + ABB + EFAH	30.02	43.32	20.96	31.07	35.58	59.15	48.32

**Table 2:** Ablation experiments on other one-stage detectors.

Models	mAP	mAP_50_	mAP_*s*_	mAP_*m*_	mAP_*l*_	Flops/G	Parameters/M
YOLOv5-s	14.23	28.52	10.65	17.71	12.83	8.12	12.33
YOLOv5-s + FWB	15.22	29.41	11.24	19.05	14.45	8.13	12.34
YOLOv5-s + EFAH	15.34	28.84	12.17	19.16	13.78	8.23	12.39
YOLOv7-tiny	21.91	37.61	13.02	24.01	25.92	6.56	6.02
YOLOv7-tiny + FWB	23.43	38.85	14.66	25.32	27.67	6.57	6.03
YOLOv7-tiny + EFAH	22.12	38.26	13.68	23.65	27.09	6.67	6.12
YOLO X-s	23.22	40.31	14.51	25.06	27.52	13.32	8.94
YOLO X-s + FWB	23.54	40.82	14.76	25.67	27.61	13.33	8.94
YOLO X-s + ABB	24.76	41.76	15.25	26.23	30.27	47.92	38.56
YOLO X-s + EFAH	23.91	41.12	14.65	25.42	28.47	15.80	9.83
SSD300	21.22	39.04	8.52	21.95	28.61	30.58	24.15
SSD300 + FWB	22.06	40.12	9.26	23.23	29.47	30.58	24.15
SSD300 + EFAH	21.63	39.37	8.63	22.67	29.09	44.72	56.23

**Table 3:** Ablation experiments on two-stage detectors.

Models	mAP	mAP_50_	mAP_*s*_	mAP_*m*_	mAP_*l*_	Flops/G	Parameters/M
Cascade R-CNN	27.23	40.95	17.12	27.26	34.01	14.27	11.14
Cascade R-CNN + FWB	27.12	41.85	18.06	27.62	34.72	14.28	11.14
Cascade R-CNN + EFAH	27.63	41.66	18.45	28.47	35.14	58.52	48.09
Faster R-CNN	25.90	41.61	17.72	25.94	33.04	236.00	69.16
Faster R-CNN + FWB	26.03	41.52	19.04	27.16	32.77	236.00	69.17
Faster R-CNN + EFAH	26.32	41.74	18.12	26.55	32.82	236.00	69.38
Deformable-DETR	27.92	44.75	15.92	29.93	30.36	208.00	41.36
Deformable-DETR + FWB	28.52	45.24	16.95	30.31	30.38	208.00	41.38
Deformable-DETR + EFAH	28.32	45.02	16.73	30.44	31.65	231.00	41.63

**Table 4:** Comparison experiments.

Models	Backbone	mAP	mAP_50_	mAP_*s*_	mAP_*m*_	mAP_*l*_	Flops/G	Parameters/M
YOLOv5-s	YOLOv5CSPDarknet	14.23	28.52	10.65	17.71	12.83	8.12	12.33
YOLOv7-tiny	E-ELAN	21.91	37.61	13.02	24.01	25.92	6.56	6.02
YOLO X-s	YOLOXCSPDarknet	23.22	40.31	14.51	25.06	27.52	13.32	8.94
SSD300	SSDVGG	21.22	39.04	8.52	21.95	28.61	30.58	24.15
YOLOv8-s	YOLOv8CSPDarknet	28.51	42.02	18.93	28.93	34.01	14.27	11.14
YOLOv10-n	CSPDarknet	29.1	42.3	19.9	30.1	35.2	8.25	2.69
Faster R-CNN	ResNet-50	25.90	41.6	17.7	25.9	30.3	208.00	41.36
Cascade R-CNN	ResNet-50	27.23	40.95	17.12	27.26	33.04	236.00	69.16
Deformable DETR	ResNet-50	27.9	44.7	15.9	29.9	35.4	193.00	40.10
Ours	CSPDarknet	30.01	43.32	20.96	31.07	35.58	59.15	48.32

## Data Availability

You can access and obtain the data through the following links: Code repository for YOLO-OD: https://github.com/jjking00/YOLO-OD (accessed on 21 November 2024). Dataset: https://aistudio.baidu.com/datasetdetail/198589 (accessed on 21 November 2024). We confirm that the code and dataset will remain publicly accessible to ensure reproducibility and verification of the experimental results.

## Abbreviations

The following abbreviations are used in this manuscript:

MDPI	Multidisciplinary Digital Publishing Institute
DOAJ	Directory of open access journals
TLA	Three letter acronym
LD	Linear dichroism
